# Initial ovarian sensitivity index predicts embryo quality and pregnancy potential in the first days of controlled ovarian stimulation

**DOI:** 10.1186/s13048-020-00688-7

**Published:** 2020-08-17

**Authors:** David Camargo-Mattos, Uziel García, Felipe Camargo-Diaz, Ginna Ortiz, Ivan Madrazo, Esther Lopez-Bayghen

**Affiliations:** 1 Clinical Research, Instituto de Infertilidad y Genética México SC, Ingenes, México City, Mexico; 2 Departamento de Toxicología, Centro de Investigación y de Estudios Avanzados del Instituto Politécnico Nacional (CINVESTAV-IPN), Avenida Instituto Politécnico Nacional 2508, San Pedro Zacatenco, 07360 México City, Mexico

**Keywords:** Poor ovarian response, Ovarian sensitivity index, Antral follicle count, In vitro fertilization, Follicle-stimulating hormone

## Abstract

**Background:**

To determine if a modified ovarian sensitivity index (MOSI), based on initial follicular measurements and the initial follicle-stimulating hormone (FSH) dose, can predict the production of high-quality embryos for successful implantation during in vitro fertilization (IVF).

**Methods:**

This study consisted of two phases: 1) a retrospective study and 2) a prospective observational study. For the first phase, 363 patients charts were reviewed, of which 283 had embryos transferred. All women underwent a standardized antagonist-based IVF protocol. At the first follow-up (Day 3/4), the number and size of the follicles were determined. MOSI was calculated as ln (number follicles (≥6 mm) × 1000 / FSH initial dose). Afterward, the number and quality of the ova, embryo development, and the number and quality of the blastocysts were determined. Embryo implantation was confirmed by β-hCG. For the second phase, 337 IVF cycles were followed to determine MOSI’s accuracy.

**Results:**

MOSI could predict the production of ≥4 high-quality embryos by Day 2 (AUC = 0.69, 95%CI:0.63–0.75), ≥2 blastocysts (AUC = 0.74, 95%CI:0.68–0.79), and ≥ 35% rate of blastocyst formation (AUC = 0.65, 95%CI:0.58–0.72). Using linear regression, MOSI was highly associated with the number of ova captured (β = 5.15), MII oocytes (β = 4.31), embryos produced (β = 2.90), high-quality embryos (β = 0.98), and the blastocyst formation rate (β = 0.06, *p* < 0.01). Using logistic regression, MOSI was highly associated with achieving ≥4 high-quality embryos (odds ratio = 2.80, 95%CI:1.90–4.13), ≥2 blastocysts (odds ratio = 3.40, 95%CI:2.33–4.95), and ≥ 35% blastocysts formation rate (odds ratio = 1.96, 95%CI:1.31–2.92). This effect was independent of age, BMI, and antral follicle count. For implantation, MOSI was significantly associated with successful implantation (odds ratio = 1.79, 95%CI:1.25–2.57). For the prospective study, MOSI was highly accurate at predicting ≥6 high-quality embryos on Day 2 (accuracy = 68.5%), ≥6 blastocysts (accuracy = 68.0%), and a blastocyst formation rate of ≥35% (accuracy = 61.4%).

**Conclusion:**

MOSI was highly correlated with key IVF parameters that are associated with achieved pregnancy. Using this index with antagonist cycles, clinicians may opt to stop an IVF cycle, under the assumption that the cycle will fail to produce good blastocysts, preventing wasting the patient’s resources and time.

## Background

Currently, one in six couples have at least one partner that suffers from infertility, the failure of achieving a viable gestation after a year of unprotected sex [1]. Over the past 40 years, assisted reproduction technologies (ART) have improved the pregnancy rates of infertile couples [2]. However, ART remains an inefficient process due to the multiple sources of variability, affecting the quantity and quality of the ova collected [2]. One of the leading causes for variability between ova cohorts is how patients respond to ovarian stimulation with follicle-stimulating hormone (FSH) or human menopausal gonadotropin (hMG) [3]. It is well documented that patients will have different responses to FSH and hMG, according to their ovarian reserve [4], age, weight, and presence of insulin resistance [3]. Depending upon the initial dose-response, the physician may adjust follow-up doses, but what remains is a lack of knowledge of the predictability of the initial dose.

One of the most critical factors for in vitro fertilization (IVF) success is to have a good follicular response, the production, and release of a sufficient number of ova (between 4 and 15) [5]. Numerous reports have demonstrated that the number of ova collected is associated with improved IVF outcomes and live birth rates [6]. Nevertheless, Rombauts et al. demonstrated that during sequential stimulation cycles, different responses could occur for each cycle to generate good embryos [7]. Therefore, a question remains, can a physician predict the potential yields of key IVF parameters, such as the number of ova collected, the number of high-quality embryos, and the number of blastocysts formed. The optimal time to answer this question would be the first measure of serum estradiol (E2) during the third or fourth day of stimulation.

The ovarian sensitivity index (OSI), developed by Biasoni et al. [8] and refined by Huber et al. [9], measures the ovarian response to the total FSH dose. However, using the total FSH dose may mislead the physician to believe that the ovarian stimulation was acceptable and too late to stop the cycle or change strategies to minimize losses. Therefore, we posit that using the initial ovarian stimulation measurements may be more accurate for determining the ovarian response. A modified OSI (MOSI) that uses the number of follicles detected on Day 3 or Day 4 during ovarian stimulation could be a better predictor. Follicles ≥6 mm on Day 3 or Day 4 of stimulation have been shown to have the highest potential to end up as mature follicles and are more likely to produce the best ovules [10]. Therefore, this research aims to determine the predictability of obtaining good quality ova that could lead to high-quality embryos in patients undergoing IVF, using the number of follicles during Day 3 or Day 4 of ovarian stimulation with the initial FSH dose.

## Methods and materials

### Study population

This study was performed in 2 phases: 1) a retrospective study that examined the association between MOSI and certain IVF parameters and 2) a prospective observational study to assess the effectiveness of MOSI. Since we are testing MOSI as a potential tool for IVF, we utilized a general IVF population, and patients would only be excluded for particular circumstances. For the retrospective study, a database with de-identified, non-coded data from women at the Instituto de Infertilidad y Genética SC, Ingenes, Mexico, was obtained (May 2018 to December 2018). Data were from women attending the clinic and underwent a standardized IVF protocol, using their own ova (no donor ova). Here, at our clinic, 80% of IVF cycles are performed with the antagonist protocol; therefore, only women that underwent an antagonist protocol were considered. There was no restriction on sperm source. The inclusion criteria were as followed: IVF cycles with controlled ovarian stimulation using the antagonist protocol; the patient’s own ova were used; had between one and three embryos transferred, and a written and signed consent allowed us to use their data. Patients were excluded if during the previous 6 months, they had any hormonal treatment, were diabetic, had a high-risk of ovarian hyperstimulation syndrome, or if their IVF cycle resulted in an ectopic pregnancy. All patients with donated ova or incomplete data were also excluded. For the prospective study, another cohort was collected from January 2019 to January 2020 for testing the efficiency of MOSI. The same inclusion and exclusion criteria for the retrospective study were used for this study. This cohort consisted of 337 IVF cycles.

### Ovarian stimulation protocol

Ovarian stimulations were performed by a group of 27 doctors under a unified protocol, reviewed by the Ingenes Quality Control Group of Medical Indicators (IQCG-IMED 2019). This internal entity reviews the Institute’s performance for indicators of medical diagnoses, embryonic development, transfer results, and pregnancy rates to detect areas of opportunity and correct errors, based on the SART parameters [11].

All patients were subjected to a controlled ovarian stimulation for 10 days with gonadotropin-releasing hormone antagonists. For each patient, the ovarian reserve was determined by the antral follicle count (AFC) using ultrasound. Anti-Müllerian hormone (AMH) levels were not determined as we had noticed the high variability between lab results as well as the high costs the patients would endure. AFC was performed, and based on the patient’s BMI, age, and size of the antral follicles, the initial FSH dose was determined, which remained unchanged for the first three to four days. Ovarian response was assessed measuring serum E2 levels, and follicular development was evaluated by ultrasound examination. To adjust the FSH dose, ultrasonographic follow-ups were programmed on alternate days according to patient availability, serum E2, and progesterone levels, and according to the Institute’s clinical criteria. Ultrasound measurements were made as an average of two perpendicular measurements of each of the follicles observed, including the follicle wall.

Follicular puncture for oocyte collection was performed under general anesthesia at the end of hormonal stimulation (10–14 days). Oocyte retrieval was conducted 36 h after ovulation was induced with human chorionic gonadotropin (hCG; 10,000 IU Choragon, Ferring Pharmaceuticals, or 6500 IU Ovidrel, Merck Laboratories). Transvaginal ultrasound guidance was used to locate mature follicles, and 3–5 ml of follicular fluid containing the oocytes were extracted using a specialized suction system. Samples were analyzed using a stereoscopic microscope in order to locate the oocytes, which were kept at 37.5 °C in an atmosphere of 8.3% CO_2_ until fertilization. An embryologist monitored and recorded the information about fertilization, embryo development, embryo morphology, transfer, and pregnancy for each oocyte. The number and quality of the retrieved oocytes were assessed using their morphological parameters (granulosa expansion, oocyte maturity (MI, MII, and VG), quality of the cytoplasm, zona pellucida, and polar body).

Embryo quality was assessed on Day 2 of embryonic development for indicators of high-quality embryos (symmetrical, consisting of 4 cells, and < 10% fragmentation). Embryonic morphological parameters evaluated were weighed into a matrix to rate each oocyte-embryo, with the sum of the values rated on a scale of 0 (low quality) to 12 (high quality). Selection for embryo transfer was made on Day 3 or Day 5 of development according to the morphological assessment, using the criteria established by the Istanbul Consensus Workshop on Embryo Assessment [12]. Our protocol defines that with < 4 good cleavage-stage embryos, the transfer is performed on Day 3. The highest quality embryos were transferred. β-hCG values confirmed clinical pregnancy (> 10 mUI/ml on Day 14). The number of embryos transferred was determined by the number of high-quality embryos achieving full development, patient results from previous attempts, and the opinion of the clinician.

### IVF outcome measurements

An embryologist monitored and recorded all information about ovarian volume, AFC, the number of ova captured and MII ova, serum E2 levels, embryonic quality (determined by the number of high-quality embryos on Day 2 of embryonic development) and their rate (compared to the total number of ova captured), as well as the number embryos able to reach a blastocyst stage on Day 5 of embryonic development and the rate of ovules that generated blastocysts. AFC was defined as the total number of follicles measuring between 2 and 10 mm in diameter that were observed during the early follicular phase by transvaginal ultrasound.

### OSI and MOSI

OSI was calculated as the total oocytes captured divided by the total FSH dose multiplied by 1000, whereas the MOSI was calculated as the total number of follicles on Day 3 or Day 4 (≥6 mm) divided by the initial dose of FSH multiplied by 1000. Due to the presence of a skewed distribution, both indices were ln-transformed. Using the method described in Huber et al., the cutoff levels for poor, normal, and high responders were determined [9]. Patients that were classified as a potential poor or high responders were explained the potential outcomes associated with the classification by the physician. Afterward, the decision to continue with the cycle was given by the patient without severe objection by the physician.

### Sample size estimation

At Ingenes, during 2018, 1447 IVF cycles were performed. Assuming a standard deviation of 31.98 and using a 95% confidence level with a maximum accepted error of 5%, the optimal sample size of 304 cycles was calculated using the PS Power and Sample Size Calculations software for MS Windows, version 3.0.11 [13].

### Statistics

The primary outcome was the association between MOSI and the number of blastocysts and the number of high-quality embryos, whereas the secondary outcomes were the number of ova captured and the number of MII ova. All analyses were performed using Statistical Package for the Social Sciences version 26 (SPSS, IBM Corp., Armonk, NY, USA). Data are presented as either mean ± standard deviation, frequencies, or percentages. The normality of continuous data was determined using the Shapiro-Wilk test. To graphically assess the association between MOSI and the IVF parameters, we used the Generalized Additive Model to plot the variables, whereas, due to the shape of the plot, linear regression was used to quantify the association. Multivariate logistic regression was used to determine the predictability of MOSI for the IVF parameters, whereas Receiver Operator Characteristic (ROC) curves were used to compare the predictability between OSI and MOSI for IVF parameters using the Hanley and McNeil method [14]. ROCs were used to determining possible cutoffs using the Youden Index (Sensitivity + Specificity − 1). Cohen’s kappa (κ) was calculated to determine the inter-assay agreement. A *p*-value < 0.05 (two-tailed) was considered significant.

## Results

### Characteristics of the study population

**Fig. 1:**
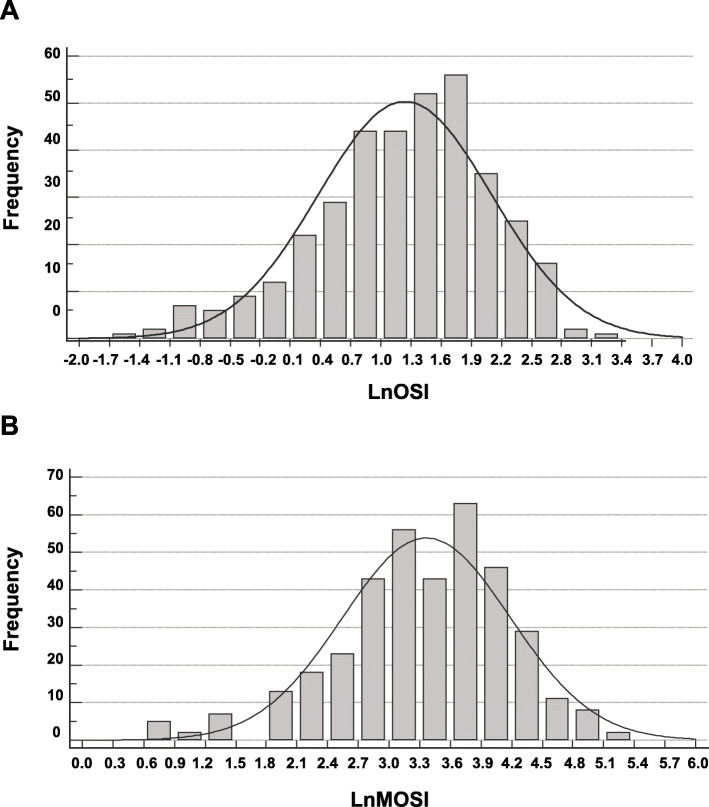
Ln-transformed distribution of (**a**) ovarian sensitivity index (OSI) and (**b**) the modified OSI (MOSI) of the study population

During the collection time, data from 947 patients (1447 cycles) were examined, of which 44.8% of these cycles were performed with donated ova and were excluded. 523 patients were identified as having IVF cycles with their own ova. One hundred sixty patients were eliminated because their data were incomplete, leaving 363 patients for this study. Only 283 patients had between one and three embryos transferred, which means that 80 cycles failed to produce a viable embryo (blocking), had aneuploid embryos, had the embryos frozen, or the patient elected not to continue with IVF. The cohort characteristics are summarized in Table 1. The main reasons for IVF were a low response, advanced age, or PCOS. The distribution of MOSI was non-parametric and was ln transformed (Fig. 1). When classifying the patients as poor, normal, or high responders, there was a 78% agreement between the OSI and MOSI (a moderate agreement, κ = 0.447, *p* < 0.001, data not shown).

**Table 1:** Patient characteristics

Variable	Value ^a^
Sample size (n)	363
Age (years)	36.1 ± 4.6 (19–45)
BMI (kg/m^2^)	25.9 ± 4.3 (18.8–38.9)
Infertility factor (n,%)	
None indicated/normal	33, 9.1%
Anovulation	8, 2.2%
Low response	80, 22.0%
Advanced age	83, 22.9%
Endometriosis	34, 9.4%
PCOS	73, 20.1%
Tubal factor	35, 9.6%
Other	17, 4.7%
FSH (IU/L)	
Initial	303 ± 78 (75–450)
Total	3104 ± 992 (75–6000)
Antral follicle count (n)	12.0 ± 8.3 (0–46)
Day 3–4 follicle count (n)	
< 6 mm	3.2 ± 3.9 (0–40)
6 to 8 mm	5.1 ± 3.9 (0–22)
> 8 mm	5.1 ± 4.2 (0–26)
OSI-modified (MOSI)	
Score	38.6 ± 28.5 (2.2–193.3)
Ln transformed	3.4 ± 0.8 (0.8–5.3)
Ova captured (n)	12.7 ± 8.0 (1–63)
MII ova (n)	10.6 ± 7.0 (0–39)
MII/Ova captured (%)	82.7 ± 17.0 (0.0–29.0)
Ovarian sensitivity index (OSI)	
Score	4.7 ± 3.7 (0.2–22.7)
Ln transformed	1.2 ± 0.9 (− 1.6–3.1)
Total embryos on Day 2 (n)	7.1 ± 5.3 (0–29)
High-quality embryos (n)	2.6 ± 2.7 (0–15)
High-quality embryos/Ova captured (%)	21.5 ± 19.3 (0–100)
Blastocyst (n)	2.5 ± 3.2 (0–16)
Blastocyst /Ova captured (%)	16.1 ± 18.7 (0–100)

^a^Values, unless indicated otherwise, are mean ± standard deviation (minimum-maximum)

### OSI and MOSI predict IVF parameters in infertile women from Central Mexico

**Fig. 2:**
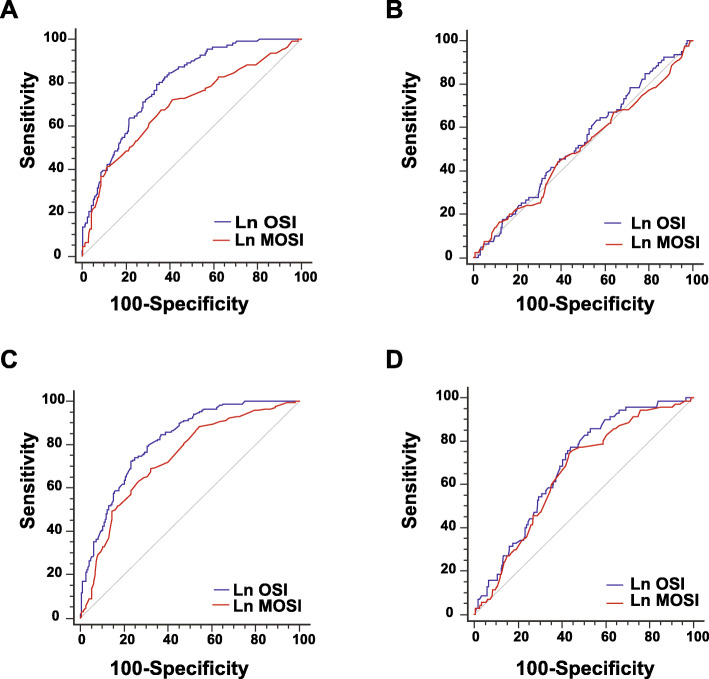
The predictability of ovarian sensitivity index (OSI) and modified ovarian sensitivity index (MOSI) for in vitro fertilization outcomes. Receiver operating characteristic curves were constructed to determine if OSI (blue line) or the MOSI (red line) can predict **a** ≥ 4 high-quality embryos, **b** a rate ≥ 35% for the number of high-quality embryos/the number of ova captured, **c** ≥ 2 blastocyst, and **d** a rate ≥ 35% for the number of blastocysts/the number of ova captured. Comparison of Area Under Curve between predictors was performed using SPSS v26

We compared the efficiency of OSI and MOSI to predict IVF parameters using ROCs. For producing ≥4 high-quality embryos by Day 2, OSI was superior to MOSI (OSI: AUC = 0.79, 95%CI: 0.75–0.84, *p* < 0.001 versus MOSI: AUC = 0.69, 95%CI: 0.63–0.75, *p* < 0.001, p_comparison_ < 0.001, Fig. 2a). Interestingly, neither index could predict a ≥ 35% rate of high-quality embryos per the total number of ova captured (OSI: AUC = 0.47, 95%CI: 0.40–0.54, *p* = 0.407 versus MOSI: AUC = 0.50, 95%CI: 0.43–0.58, *p* = 0.964, p_comparison_ = 0.239, Fig. 2b). For producing ≥2 blastocysts, OSI was again superior to MOSI (OSI: AUC = 0.82, 95%CI: 0.77–0.86, *p* < 0.001 versus MOSI: AUC = 0.74, 95%CI: 0.68–0.79, *p* < 0.001, p_comparison_ < 0.001, Fig. 2c). However, both indices could predict a ≥ 35% rate of blastocyst formation per the total number of ova captured and neither indices were superior (OSI: AUC = 0.69, 95%CI: 0.63–0.75, *p* < 0.001 versus MOSI: AUC = 0.65, 95%CI: 0.58–0.72, *p* < 0.001, p_comparison_ = 0.244, Fig. 2d). These results suggest that OSI and MOSI can predict certain IVF parameters, but neither index is better at predicting the blastocyst formation rate.

### MOSI predicts high-quality ovules and embryos

**Fig. 3:**
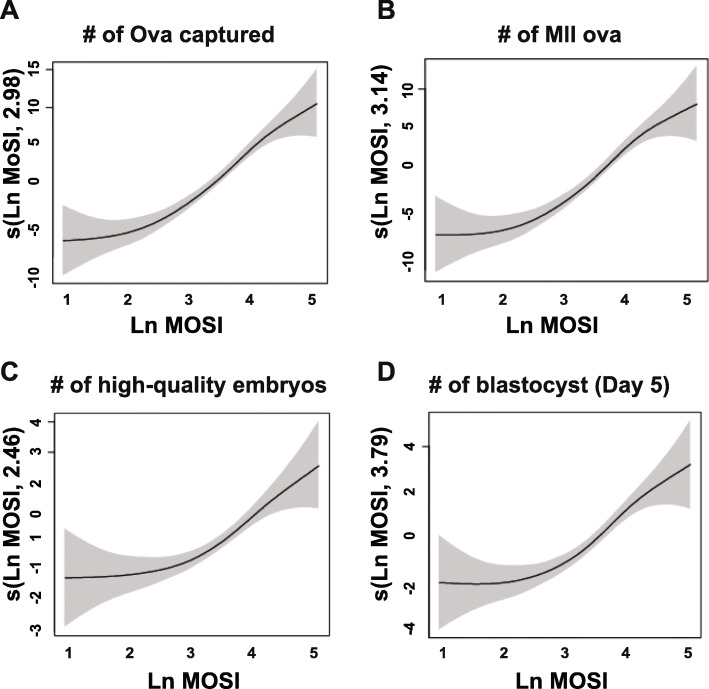
Fitted values for **a** number of ova captured, **b** the number of MII ova, **c** number of high-quality embryos, and **d** number of blastocysts on Day 5 according to modified ovarian sensitivity index (MOSI) using Generalized additive model with smoothing splines

Graphic representation of MOSI and the total number of ova captured (Fig. 3a), MII oocytes (Fig. 3b), high-quality embryos (Fig. 3c), and blastocysts (Fig. 3d) demonstrate a positive correlation between MOSI and the IVF parameters. The strength of the association between MOSI and the IVF parameters was measured by linear regression (Table 2). MOSI was highly associated with the number of ova captured (5.15 ova per a MOSI unit), the number of MII oocytes (4.31 MII oocytes per a MOSI unit), the number of total embryos produced (2.90 embryos per a MOSI unit), the number of high-quality embryos (0.98 embryos per a MOSI unit), the number of the blastocyst (1.43 embryos per a MOSI unit), and the blastocyst formation rate (6% per a MOSI unit).

**Table 2:** Linear regression results

Category	Correlation coefficient	Beta	Standard Error	*P*-value
Ova captured	0.510	5.15	0.46	< 0.001
MII ova	0.493	4.31	0.40	< 0.001
MII ova/ Ova capture rate	0.001	0.00	0.01	0.979
Embryos	0.445	2.90	0.31	< 0.001
High-quality embryos	0.287	0.98	0.17	< 0.001
High-quality embryos/Ova capture rate	0.014	0.00	0.01	0.795
Blastocyst	0.356	1.43	0.20	< 0.001
Blastocyst/ Ova capture rate	0.265	0.06	0.01	< 0.001

Using logistic regression, we determined the ability to achieve key goals associated with better IVF outcomes (Table 3). MOSI was highly associated with achieving ≥4 high-quality embryos and ≥ 2 blastocysts, and this effect was independent of age and BMI (model 1) and AFC (model 2). Moreover, achieving a rate of ≥35% blastocysts per the total number of ova captured were also significantly associated with MOSI and independent of age, BMI, and AFC. For embryo implantation, MOSI was significantly associated with implantation; however, this effect was lost when AFC was considered. Lastly, another cohort was collected 1 year later. Using the ROCs (Fig. 2), cutoffs were calculated using the highest Youden Index score. These cutoff values were used to assess the test accuracy. For each parameter, MOSI resulted in at least 60% accuracy (Table 4). Moreover, the Negative Predictive Value (NPV) was higher than 80% for each parameter.

**Table 3:** Logistic regression

Category	Crude model	Model 1 ^a^	Model 2 ^b^
≥4 High-quality embryos	2.80 (1.90–4.13), < 0.001*	2.47 (1.64–3.70), < 0.001*	1.88 (1.20–2.95), 0.006*
≥35% High-quality embryos/ova captured	0.97 (0.69–1.37), 0.876	0.94 (0.65–1.34), 0.717	1.05 (0.69–1.58), 0.832
≥2 blastocyst embryos	3.40 (2.33–4.95), < 0.001*	3.54 (2.36–5.33), < 0.001*	3.30 (2.11–5.17), < 0.001*
≥35% blastocyst embryos /ova captured	1.96 (1.31–2.92), 0.001*	2.01 (1.30–3.10), 0.002*	1.94 (1.18–3.18), 0.009*
Implantation (pregnancy) ^c^	1.79 (1.25–2.57), 0.002*	1.53 (1.05–2.23), 0.028*	1.49 (0.97–2.30). 0.070

Values are Odds ratio (95% confidence interval), *p*-value. * indicates a significant result (*p* < 0.05, two-tailed)

Crude model = odds ratios are no adjusted

^a^ Model 1 = odds ratios are adjusted by age and BMI. Note: 48 patients (41 implanted cycles) were missing BMI information

^b^ Model 2 = odds ratios are adjusted by age, BMI, and AFC

^c^ Note: only 283 patients had their embryos transferred

**Table 4:** Test efficiency of the Modified Ovarian Sensitivity Index for in vitro fertilization parameters (additional cohort)

Category	MOSI score	All data (*n* = 337)				Only normo-responders (*n* = 255)			
		Prevalence	Test accuracy	PPV	NPV	Prevalence	Test accuracy	PPV	NPV
High-quality Embryo on Day 2 (≥6)	2.1	27.3%	33.2%	28.6%	88.5%	27.1%	27.1%	27.1%	N/A
	2.6		43.4%	32.0%	93.5%		35.7%	29.4%	95.8%
	3.1		54.3%	36.0%	89.6%		50.2%	33.7%	88.3%
	**3.6**		**68.5%**	**45.2%**	**86.4%**		**69.0%**	**45.1%**	**85.0%**
	4.1		71.8%	47.1%	76.2%		73.3%	57.1%	73.8%
	4.6		71.5%	16.7%	72.5%		72.9%	N/A	72.9%
Blastocysts (≥6)	2.0	35.9%	39.5%	36.9%	80.0%	35.7%	35.7%	35.7%	N/A
	2.5		44.8%	39.1%	89.5%		35.7%	35.7%	N/A
	3.0		54.9%	43.4%	81.4%		49.0%	39.8%	76.6%
	**3.5**		**68.0%**	**54.0%**	**80.7%**		**66.3%**	**52.1%**	**78.3%**
	4.0		69.4%	64.1%	70.7%		68.2%	75.0%	67.7%
	4.5		65.3	70.0%	65.1%		64.3%	N/A	64.3%
Rate of blastocyst/ova captured (≥35%)	2.0	18.1%	22.8%	18.6%	90.0%	18.0%	18.0%	18.0%	N/A
	2.5		28.2%	19.7%	94.7%		18.0%	18.0%	N/A
	3.0		43.0%	22.1%	91.2%		37.6%	20.4%	89.1%
	**3.5**		**61.4%**	**28.6%**	**91.5%**		**62.0%**	**28.2%**	**90.6%**
	4.0		74.8%	31.3%	85.0%		79.6%	35.0%	83.4%
	4.5		80.7%	30.0%	82.3%		82.0%	N/A	82.0%
	4.8		81.3%	0%	81.8%		82.0%	N/A	82.0%

*Abbreviations*: *MOSI* modified ovarian sensitivity index, *NPV* negative predictive value, and *PPV* Positive predictive value

Bold values are the results based on the cutoff using the highest Youden index based on the Receiver-operating characteristic curve

## Discussion

Ovarian reserves are shown to decrease with a woman’s age [4, 15], but women of the same age can respond differently to FSH stimulation [10, 15]. When a patient is undergoing IVF, the first time point in which the ovarian response can be measured is between three to 4 days after the initial FSH dose. The question remains, can this initial response determine the feasibility of the IVF cycle to produce viable embryos for implantation? Indeed, the response on Day 3 or 4 after stimulation did highly associate with ova production and of the number of embryos that developed.

An inadequate response to FSH has been correlated with diminished ova production, which significantly affects the possibility of an ideal gestation in ART [16]. It is known that in high-response cycles when there are too many follicles (> 25 ova), the number of genetic alterations is augmented [17, 18]. When < 6 ova are obtained, there are less high-quality ova, and the IVF cycle is more likely to produced sub-optimal embryos [4]. Here, MOSI did correlate with the number of ova isolated as well as the MII oocytes count (Fig. 3). Therefore, this index does present with the ability to identify IVF cycles in which the cycle may fail to produce a sufficient number of ova or an excessive amount. Thus, MOSI could aid physicians in deciding to continue a cycle.

Huber et al. described the capabilities of OSI, in which even the possibility of pregnancy was predicted, but with the inconvenience of utilizing this index only after the ova were collected and the ovarian stimulation-protocol was completed [9]. This has an unfortunate consequence, given the fact that the main costs of an IVF cycle occur once the ova are collected. If the IVF cycle results in poor-quality ova, there is no possibility of stopping the procedure in time to restart the cycle to achieve a better stimulation [19]. Hassan et al. described the follicular sensitivity index (FSI). Although this model predicts the possibility of achieving a pregnancy, it is necessary to have finished the ovarian stimulation, as it uses total FSH dose. The FSI cannot predict from the beginning of the stimulation if high-quality embryos will be obtained [20]. Again, the clinician would try to achieve clinical pregnancy with embryos whose quality, in some cases, is not sufficient. Here, MOSI could determine, with similar efficiency as OSI and FSI, if clinical pregnancy was achieved, but MOSI is doing it at an early phase.

Currently, depending on the IVF laboratory, it has been determined that only between 24 and 50% of the ova obtained will develop into a viable blastocyst that can be transferred [21]. Another factor for having viable blastocysts for transfer is if there are at least 4 high-quality embryos on Day 2, which is correlated with ova capture. This has led to most fertility facilities to try to obtain at least 6 ova during the harvesting step. Numerous studies have examined how the FSH dose affects the follicles observed by ultrasound and the synchrony in their growth [10, 22, 23]. The changes in serum E2 and progesterone levels as well as ultrasound measurements of folliculometry [24] can serve as tools to predict the correct evolution of the stimulation; nevertheless, they remain insufficient. MOSI highly correlated with ova captured and with having at least 4 high-quality embryos on Day 2. Therefore, MOSI can be utilized as an additional and early measure to improve IVF outcomes, as the stimulation can be canceled, giving the patient a better chance with another stimulation protocol, potentially yielding a better outcome.

Here, using either OSI or MOSI, we identified 9.9% of the IVF cycles as poor responders (data not shown). In poor responders, dehydroepiandrosterone (DHEA) promotes early follicular development [25], a higher number of oocytes at harvesting, and Day 3 embryos with improved clinical pregnancy rates as well as live birth rates [26, 27]. DHEA, coupled with MOSI, could indicate if a previously identified poor responder will yield improved IVF parameters; however, future studies are required. Another concern for poor responders is the premature rise of LH. Even though gonadotropin-releasing hormone analogs can prevent the premature rise [28], the potential LH surge remains [29]. When follicle recruitment during the luteal phase is performed using luteal phase ovarian stimulation (LPOS) [30], LPOS did augment oocytes pick-up and the number of Day 3 embryos, but there was no difference in the pregnancy rate [31]. Again, LPOS, coupled with MOSI, could be another option for poor responders.

To reiterate, one of the key prognostic factors for IVF is collecting the optimal number of oocytes, 8 to 14, and many researchers are developing indices to predict the optimal initial FSH dose to achieve this goal. For example, La Marca et al. developed a normogram, based on the patient’s age as well as serum AMH and FSH levels that was to indicate the optimal initial FSH dose [32], and when compared to local protocols, it was determined that ~ 13% of patients received a dose that was either suboptimal or excessive [33]. With high predictability, this index led to improve IVF parameters. MOSI would also complement this index, indicating if the initial dose was sufficient. However, women with PCOS remain under-evaluated and this is further confounded when considering the extreme variability of severity associated with PCOS and its associated comorbidities: insulin resistance, depression, hypertension, etc. [34]. Insulin resistance significantly augments cancellation rates and diminishes conception rates in PCOS women during IVF [35, 36]. Therefore, any index to be used on PCOS women, such as MOSI, the La Marca normogram, or POSEIDON, should be evaluated for insulin resistance’s effect on FSH stimulation. MOSI determines the effectiveness of the initial FSH dose; however, it could be inaccurate for severe insulin-resistant women, independent of PCOS. Another confounder would be the treatment with Metformin or any insulin-sensitizing agent. Metformin was shown to improve insulin resistance as well as the number of oocytes collected [37]; therefore, future studies would be required to determine the effect insulin resistance has on MOSI and other indices with PCOS or insulin-resistant women at predicting ovarian response and IVF outcomes.

This study has a few limitations. First, even though we represented the data using Generalized Additive Models, the associations were assessed with linear regression. The model would fit better using other possibilities; however, the majority of the graph appeared linear. Therefore, the association can be assessed over a limited range. Second, the cohort was made of women suffering from many different etiologies of infertility. It is possible that MOSI could be affected by the cause of infertility. Third, the subsequent FSH doses varied significantly from a similar initial dose. This would affect the associations; however, the change in subsequent FSH doses is dependent upon many factors.

## Conclusions

Here, MOSI, an index based on the initial FSH dose and follicle development, allows the prediction of ovarian stimulation outputs and if the antagonist-based IVF cycle will lead to the development of high-quality embryos. This will allow a clinician to be able to decide to continue with the cycle or to change to a new stimulation in a subsequent cycle. The importance of this work lies in the high costs associated with reaching a follicular aspiration procedure with poor ova quality. Lastly, this study lays the groundwork for future prospective studies, in which MOSI could be used to modify FSH concentrations during an antagonist-based ovarian stimulation, promoting optimal ova production without leading to the development of ovarian hyperstimulation syndrome or other adverse effects.

## Data Availability

The datasets used and analyzed for the current study are available from the corresponding author on reasonable request.
